# Potential Inhibitors of Monkeypox Virus Revealed by Molecular Modeling Approach to Viral DNA Topoisomerase I

**DOI:** 10.3390/molecules28031444

**Published:** 2023-02-02

**Authors:** Xiaopeng Hu, Sanqi An, Jiemei Chu, Bingyu Liang, Yanyan Liao, Junjun Jiang, Yao Lin, Li Ye, Hao Liang

**Affiliations:** Biosafety Level-3 Laboratory, Life Sciences Institute & Guangxi Collaborative Innovation Center for Biomedicine, Guangxi Medical University, Nanning 530021, China

**Keywords:** monkeypox virus, DNA topoisomerase I (TOP1), natural products, rosmarinic acid, molecular dynamics simulation, Surface Plasmon Resonance (SPR), enrichment analyses

## Abstract

The monkeypox outbreak has become a global public health emergency. The lack of valid and safe medicine is a crucial obstacle hindering the extermination of orthopoxvirus infections. The identification of potential inhibitors from natural products, including Traditional Chinese Medicine (TCM), by molecular modeling could expand the arsenal of antiviral chemotherapeutic agents. Monkeypox DNA topoisomerase I (TOP1) is a highly conserved viral DNA repair enzyme with a small size and low homology to human proteins. The protein model of viral DNA TOP1 was obtained by homology modeling. The reliability of the TOP1 model was validated by analyzing its Ramachandran plot and by determining the compatibility of the 3D model with its sequence using the Verify 3D and PROCHECK services. In order to identify potential inhibitors of TOP1, an integrated library of 4103 natural products was screened via Glide docking. Surface Plasmon Resonance (SPR) was further implemented to assay the complex binding affinity. Molecular dynamics simulations (100 ns) were combined with molecular mechanics Poisson–Boltzmann surface area (MM/PBSA) computations to reveal the binding mechanisms of the complex. As a result, three natural compounds were highlighted as potential inhibitors via docking-based virtual screening. Rosmarinic acid, myricitrin, quercitrin, and ofloxacin can bind TOP1 with KD values of 2.16 μM, 3.54 μM, 4.77 μM, and 5.46 μM, respectively, indicating a good inhibitory effect against MPXV. The MM/PBSA calculations revealed that rosmarinic acid had the lowest binding free energy at −16.18 kcal/mol. Myricitrin had a binding free energy of −13.87 kcal/mol, quercitrin had a binding free energy of −9.40 kcal/mol, and ofloxacin had a binding free energy of −9.64 kcal/mol. The outputs (RMSD/RMSF/Rg/SASA) also indicated that the systems were well-behaved towards the complex. The selected compounds formed several key hydrogen bonds with TOP1 residues (TYR274, LYS167, GLY132, LYS133, etc.) via the binding mode analysis. TYR274 was predicted to be a pivotal residue for compound interactions in the binding pocket of TOP1. The results of the enrichment analyses illustrated the potential pharmacological networks of rosmarinic acid. The molecular modeling approach may be acceptable for the identification and design of novel poxvirus inhibitors; however, further studies are warranted to evaluate their therapeutic potential.

## 1. Introduction

Monkeypox virus (MPXV) is an emergent human pathogen, and the endemic was sporadically reported in Africa. However, it has posed as a new global outbreak since May 2022. MPXV is a double-stranded DNA virus, and it belongs to the Poxviridae orthopoxvirus family. The orthopoxviruses, including monkeypox virus, smallpox virus, vaccinia virus, cowpox virus, and buffalopox virus, infect various mammals and cause zoonosis [1]. Although MPXV is less lethal than smallpox, it is considered to be a dangerous virus, because it causes many challenges, including high prevalence, disability, and disfiguration [2]. In the past, there have been occasional outbreaks of the MPXV infection, which were mainly caused by people contacting infected rodents. Most infected cases experience fever, body aches, chills, and tiredness [3]. Patients with severe symptoms may develop a rash and cuts towards the face and hands that can spread the infection to other parts of the body. MPXV has a fatality rate of up to 10% and is thought to be more severe among children [1]. Previously, MPXV infection was mainly endemic in Western Africa [2]. However, with the number of cases outside Africa increasing rapidly in 2022, MPXV infection has led to high levels of concern. The World Health Organization (WHO) announced that the multi-country MPXV outbreak has become a global public health emergency at a conference of the International Health Regulations Emergency Committee (EC) on 23 July 2022.

More than 40 years have passed since the WHO declared the extinction of smallpox disease worldwide. The vaccination and immunization programs against smallpox have been halted since 1980. It is estimated that half of the general population is currently unvaccinated against smallpox [3]. Therefore, the general population is highly susceptible to outbreaks caused by the release of the smallpox virus. Moreover, the progressive move of zoonotic viruses from endemic reservoirs or the potential diffusion of variola virus due to bioterrorism behavior places animal poxviruses in the range of high epidemic risk pathogens. In this context, renewed interest in the discovery and exploitation of novel antiviral medicine can be applied to help mitigate the influence of future outbreaks [4].

The most effective choice for preventing orthopoxvirus infections is vaccination. However, the variability and mutation of viruses shorten the efficacious window of postexposure vaccination (from only 4 to 7 days) [5]. Moreover, complications upon vaccination, such as encephalitis, fetal vaccinia, progressive vaccinia, and eczema vaccinatum, often occur among individuals with acquired or congenital immunodeficiency [6]. Although vaccination remains critical to controlling outbreaks, the validity of antiviral medical treatment would be meaningful for individuals with vaccine contraindications and those who are infected [7].

Generally, the lack of valid and safe medicine hinders the eradication of orthopoxvirus infections. No specific treatment for MPXV has been demonstrated to date. The existing MPXV treatments mainly include nursing, symptomatic, and supportive treatment. Most existing drugs are nucleoside analogs, such as cidofovir, which inhibit virus replication. Other nucleoside analogs, such as adenozine N1-oxide (ANO) and its derivatives; N-methanocarbothymidine; or derivatives of aciklovir, peninclovir, and brivudin, have also been reported to be effective in inhibiting infection by limiting the release of progeny virions [8]. However, side effects and drug resistance limit the therapeutic potential of nucleoside analogs in response to new outbreaks of the orthopoxvirus.

Importantly, the transmission and mutation of MPXV are a high concern and priority. DNA topoisomerase I (TOP1) is a highly conserved DNA repair enzyme with a small size and low homology to human proteins, making it an attractive target for antiviral virtual screening [9]. Additionally, many enzymes are encapsidated in the orthopoxvirus. TOP1 is a crucial enzyme required for an early phase of viral transcription. Therefore, TOP1 is considered a potential antiviral target [10]. It functions by liberating the supercoiled and torsional tension towards the DNA duplex. It also introduces single-stranded breaks via transesterification at the specific site of 5′-[CT]CCTTp in the DNA duplex [9]. The scissile phosphodiesters are attacked through the catalytic tyrosine of the enzyme, providing the constitution of a DNA–(3’-phosphotyrosyl)–enzyme intermediate and the exclusion of a 5′-OH DNA strand [9,10].

Natural products including TCM have been an underexploited reservoir of novel compound candidates to combat a variety of illnesses. Numerous natural products have antiviral properties and have been tested [11]. Traditional Chinese Medicine (TCM) is rich in biological diversity and can be further developed to discover fresh compound candidates. Those therapeutic compounds are primarily prepared from heat-clearing and detoxifying herbs, which are rich in different kinds of alkaloids, terpenes, flavonoids, saponins, and carboxylic acids [11,12]. Computer-aided drug design (CADD) has become more advantageous as compared with the traditional approach of screening in reducing the dissipation of resources in terms of cost, time, and effort through markedly diminishing the quantity of compounds and filtering out hits for further HTS [12]. Here, the homology model of TOP1 of MPXV was obtained using SWISS-MODEL, and we identified potential natural product-derived MPXV inhibitory compounds via docking-based virtual screening. Molecular dynamics simulations (100 ns) were combined with molecular mechanics Poisson–Boltzmann surface area (MM/PBSA) computations to reveal the binding mechanisms of the complex. The natural product-derived compounds from Traditional Chinese Medicine (TCM) with limited side effects or complications will undoubtedly enlarge the arsenal of antiviral agents.

## 2. Results

### 2.1. Modeling the Structure of TOP1

**Figure 1:**
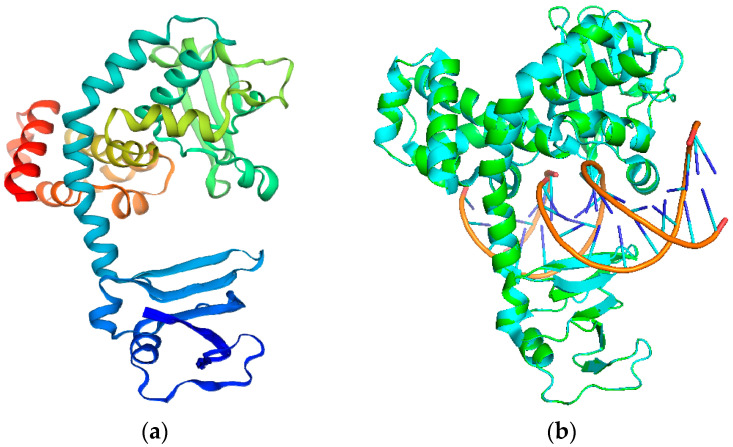
(**a**) Cartoon views of predicted 3D structures of the TOP1 from the SWISS-MODEL; (**b**) the structural alignment between the modeled TOP1 and template TOP1 (PDB ID 3IGC) from the variola virus (green—modeled TOP1; cyan—template TOP1).

We chose the protein sequence TOP1 (F1DIV3) from the UniProt database corresponding to the available experimental MPXV TOP1 model. The MPXV TOP1 sequences show only 28% were identical to the human P11387 when analyzed with the protein blast alignment tool (NCBI). Then, the amino acid sequences were submitted into SWISS-MODEL, which implemented a Basic Local Alignment Search Tool (BLAST) search to obtain reasonable templates that were identical to the target sequence. The TOP1 structures, from the variola virus or vaccinia virus (PDB ID: 3IGC, 2H7F, and 1A41) ranked top, with identical scores of 96.15%, 98.08%, 96.94%, respectively. Indeed, the sequence identity score between the template and the query exceeded 35%, indicating the model was created with high quality. Thus, the three structures were then chosen as the structural template to model MPXV TOP1 (Figure 1). Subsequently, MPXV TOP1 was verified as the best with the structure assessment (GMQE 0.93, QMEAND 0.85 ± 0.05). Therefore, MPXV TOP1 was successfully used for studies of structure-based molecular modeling.

### 2.2. Quality Evaluation of MPXV TOP1 Models

**Figure 2:**
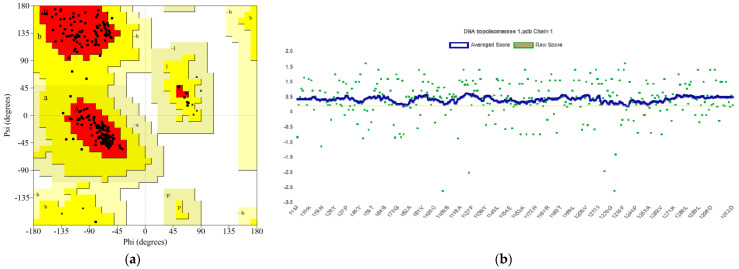
(**a**) Ramachandran plot towards TOP1 model; (**b**) results of the Verify_3D analysis of the TOP1 model.

The model was evaluated using SAVES v6.0. SWISS-MODEL had a good ERRAT. The Ramachandran plot was also procured utilizing PROCHECK, which assessed the stereochemistry of TOP1 by identifying residue-by-residue geometry and whole-structure geometry (Figure 2a) [12]. Due to the percentage of residues in the most favored (core), additionally allowed, generously allowed, and disallowed regions, the TOP1 structure was considered high quality [13,14]. The protein structure of TOP1 had 95.5%, 4.5%, 0.0%, and 0.0% towards the residues in the most favored, additionally allowed, generously allowed, and disallowed regions, respectively. Verify 3D was applied to determine the matched degree of the TOP1 model according to its amino acid sequence (Figure 2b) and a PROVE score of 95.082%. It was also forecasted by PROCHECK to have 0 faults and three passes, indicating that neither misfolded nor erroneous regions were identified.

### 2.3. Docking-Based Virtual Screening

The integrated library from the Traditional Chinese Medicine Library (L8300) and the Natural Product Library (L1400) and the classical inhibitor ofloxacin docked with the energy minimized TOP1 at a grid pocket size of 28.32 × −10.89 × 29.89 Å. Centered at (10, 12, 14), Å was set as the binding domain of TOP1. Ofloxacin is one kind of fluoroquinolone. It acts on DNA gyrase and topoisomerase IV, enzymes which, similar to human topoisomerase, prevent the excessive supercoiling of DNA during replication or transcription. By inhibiting their function, the drug inhibits normal cell division. It was demonstrated that ofloxacin and levofloxacin inhibited the viral topoisomerase activity of the vaccinia virus but not of the herpes simplex virus and influenza virus [15]. A previous report also showed that fluoroquinolone enrofloxacin inhibits DNA relaxation by Vaccinia topoisomerase I [16]. Therefore, we chose ofloxacin as a positive inhibitor compared with other natural compounds, looking at the amount of 4103 compounds that were successfully sifted against TOP1. A stringent docking score standard of −7 kcal/mol was set to choose the compounds after the virtual screening. A total of three compounds were highlighted with docking scores of less than −7 kcal/mol. A negative approach was utilized to rank the export in the order of the diminishing binding affinity. As a result, the higher the negativity, the more reasonably the candidate performed as a feasible supreme compound [17].

Rosmarinic acid was proven to have the lowest binding energy to TOP1, with a docking score of −8.207 kcal/mol. Myricitrin, quercitrin, and ofloxacin also demonstrated low docking scores of −7.599 kcal/mol, −7.322 kcal/mol, and −6.046 kcal/mol, respectively. The other compounds demonstrated a weaker binding affinity to TOP1 than the classical inhibitor ofloxacin and are excluded in the following Table 1. As a classical inhibitor, ofloxacin was demonstrated to have a docking score of −6.246 kcal/mol to TOP1 in this study. Ofloxacin with ~2 mM was reported to induce ~30% inhibition of TOP I, which was purified from vaccinia [16]. This implies that compounds with a lower docking score than ofloxacin can feasibly demonstrate significant prohibitive activities towards MPXV.

### 2.4. Characterization of TOP1–Ligand Interactions

**Figure 3:**
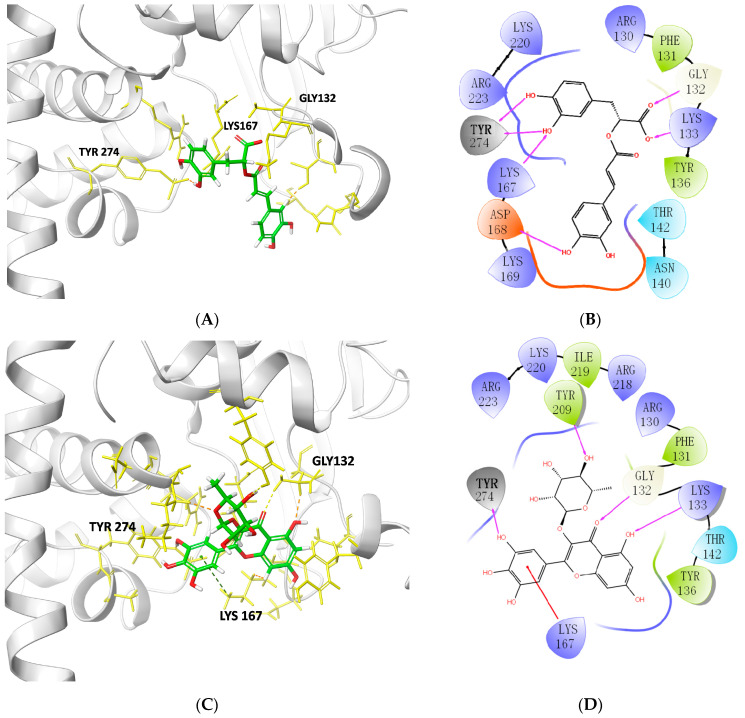

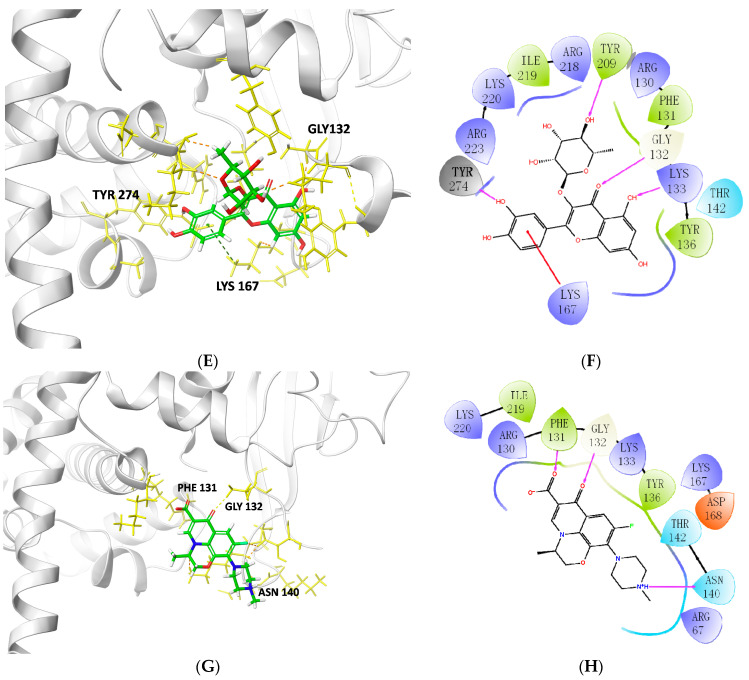
Cartoon on behalf of TOP1 targeted with: (**A**,**B**) rosmarinic acid; (**C**,**D**) myricitrin; (**E**,**F**) quercitrin; (**G**,**H**) ofloxacin. The 3D combining site was revealed as a cartoon representation, as well as the compounds, shown as sticks. The 2D one was further visualized and signed in the indicated atoms with the interaction force, including the hydrogen bond and hydrophobic contact.

Rosmarinic acid was docked into the TOP1 pocket and shaped into two hydrogen bonds with TYR274 of lengths 2.67 and 2.71 Å. Other hydrogen bonds were also found, including LYS167, GLY132, LYS133, and ASP168. It also interacted with ARG223, LYS220, ARG130, PHE131, TYR136, and THR142 via hydrophobic bonds (Figure 3A, Table 2). The interaction between myricitrin and TOP1 was created through one hydrogen bond with TYR274 (2.94 Å), as well as with TYR209, LYS167, GLY132, and LYS133. It also formed hydrophobic contacts with ARG218, ILE219, LYS220, ARG223, ARG130, PHE131, TYR136, and THR142 (Figure 3B, Table 2). Quercitrin, which docked into the TOP1 pocket, interacted with TYR209, TYR274, LYS167, LYS133, and GLY132 via hydrogen bonding and ILE219, LYS220, ARG223, ARG130, PHE131, TYR136, and THR142 via hydrophobic bonding (Figure 3C, Table 2). Quercitrin’s situation is quite close to that of myricitrin due to the similar structural types of the flavonoids. The interactions between the selected compounds and the residues may explain their high MPXV suppression. Ofloxacin targeted TOP1 via hydrogen bonds with TYR136 with bond lengths of 3.40 Å (Figure 3D, Table 2). Only one hydrogen bond was observed between ofloxacin and TOP1. This may be the critical reason for its weaker affinity compared with the above natural products. Ofloxacin also formed hydrophobic contacts with ARG67, ARG130, LYS133, TYR136, THR142, LYS167, ASP168, ILE219, and LYS220.

### 2.5. Molecular Dynamics Simulations

**Figure 4:**
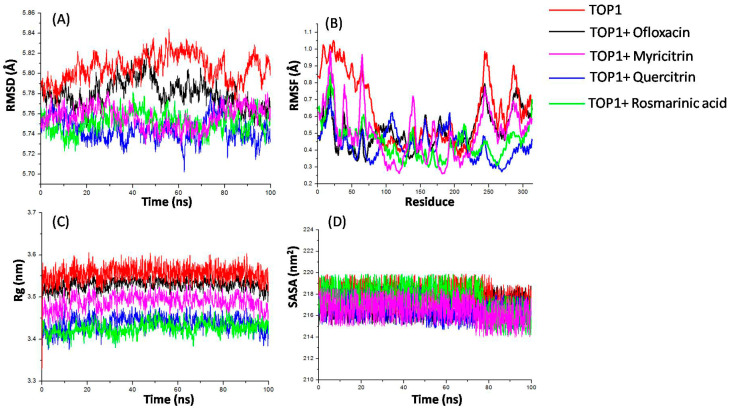
Root mean square deviation (RMSD), root mean square fluctuation (RMSF), radius of gyration (Rg), and solvent-accessible surface area (SASA) graphs of the TOP1–ligand complexes produced over a 100 ns molecular dynamics simulation. (**A**) RMSD versus time plot of TOP1–ligand complexes; (**B**) analysis of the RMSF trajectories of residues of TOP1–ligand complexes; (**C**) Rg versus time plot of TOP1–ligand complexes; (**D**) SASA versus time plot of TOP1–ligand complexes; as for the unbound protein, TOP1, TOP1+Ofloxacin, TOP1+Rosmarinic acid, TOP1+Myricitrin, and TOP1+Quercitrin are represented as red, black, purple, blue, and green, respectively.

The 100 ns MD simulations were implemented successfully on the compounds–TOP1 complexes to evaluate whether the MD simulations converged and whether they were stable. The root mean square deviation (RMSD), the root mean square fluctuation (RMSF), the radius of gyration (Rg), and the solvent-accessible surface area (SASA) were analyzed for unbound protein and protein–ligand complexes (Figure 4) [18]. The outputs indicated well-behaved systems.

TOP1 advanced to a mean of 5.7 Å until about 2 ns and then retained its balance during a period of 100 ns simulation. The RMSD plot for the complex presented the same tendency as the unbound TOP1 (Figure 4A). Interestingly, the TOP1–rosmarinic acid complex performed minimum fluctuations, maintaining an average 5.5 Å value during the period. Both the myricitrin and quercitrin complexes presented less fluctuations than ofloxacin. The TOP1–ofloxacin complex was proven to have the highest RMSD values, with an average of 5.8 Å, suggesting the most fluctuations.

RMSF discloses the elasticity of diverse sites of protein–ligand complexes [19,20]. The stability profile analysis was utilized to assess residues dedicated to the complex structural undulation. Higher RMSF values mean greater fluctuations in residues. The RMSF plots revealed that both rosmarinic acid and myricitrin caused some fluctuations in similar regions of TOP1 (Figure 4B). Fluctuations were perceived at regions from residue indices 1–75, 100–175, and 225–300. A high fluctuation was observed between residues 1–75 and 225–300, hinting they could be involved in compound binding. The outcome is consistent with the binding mode analysis above.

Rg was used to depict the characteristic of protein compactness and the folding mechanism [19]. The conformational behavior of the TOP1, TOP1+Ofloxacin, TOP1+Rosmarinic acid, TOP1+Myricitrin, and TOP1+Quercitrin systems was calculated with average Rg values of 3.44 nm, 3.48 nm, 3.43 nm, 3.53 nm, and 3.55 nm, respectively (Figure 4C). Rg values show a minor decrease when the selected compounds are bound with TOP1. The protein–ligand complexes remained stable with a stable equilibrium of Rg for 100 ns MD. A lower Rg value was observed while rosmarinic acid interacted with TOP1, indicating a higher compactness of TOP1. Therefore, the complex folding was less frequent.

SASA is a conducive parameter used to study the conformational dynamics of a protein in the surrounding solvent [19]. The average SASA values for TOP1, TOP1+Ofloxacin, TOP1+Rosmarinic acid, TOP1+Myricitrin, and TOP1+Quercitrin were calculated as 217.01 nm^2^, 217.84 nm^2^, 216.70 nm^2^, 217.61 nm^2^, and 216.5 nm^2^, respectively (Figure 4D). A lower decrease in the SASA of TOP1 during targeting with rosmarinic acid occurred, possibly due to some external residues being concealed by the protein pocket, indicating that a potential hydrophobic force was formed within the complex.

**Figure 5:**
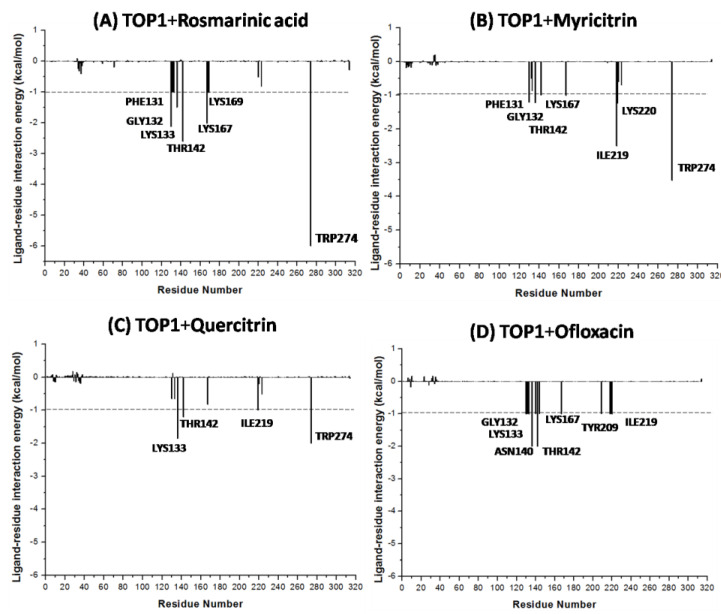
Decomposition of MM/PBSA into contributions from individual residues for (**A**) TOP1+Rosmarinic acid; (**B**) TOP1+Myricitrin; (**C**) TOP1+Quercitrin; (**D**) TOP1+Ofloxacin.

The MM/PBSA calculations revealed that rosmarinic acid had the lowest binding free energy at −16.18 kcal/mol. It was also observed that myricitrin has a binding free energy of −13.87 kcal/mol, but quercitrin had −9.40 kcal/mol, and ofloxacin had −9.64 kcal/mol (Table 2). The MM/PBSA method was also utilized to explore free binding energies using per-residue decomposition. The useful perception of meaningful interactions of crucial residues in free energy contributions was performed with decomposition. The TOP1–ligand spectra interactions presented a significant number of critical residues (PHE131, GLY132, LYS133, THR142, LYS167, LYS169, and TYR274), which contributes favorably to the binding of TOP1+Rosmarinic acid in comparison to TOP1+Ofloxacin (GLY132, LYS133, ASN140, THR142, LYS167, TYR209, and ILE219) (Figure 5A,D). Residues conferring a binding free energy greater than 1.0 kcal/mol or less than −1.0 kcal/mol were identified as critical residues for the interaction. It can further be observed from Figure 5A that TYR274 (−6.00 kcal/mol), THR142 (−2.60 kcal/mol), LYS167 (−2.02 kcal/mol), LYS133 (−1.02 kcal/mol), PHE131 (−1.00 kcal/mol), and LYS169 (−1.01 kcal/mol) contribute significantly to the binding between rosmarinic acid and TOP1, looking at the computations of MM/PBSA per-residue decomposition. Regarding the TOP1+Rosmarinic acid complex, PHE131, GLY132, LYS133, THR142, LYS167, LYS169, and TYR274 were observed to contribute individual energies beyond the −1.0 kcal/mol threshold, respectively. As for the TOP1+Myricitrin complex (Figure 5B), it was observed that PHE131, GLY132, THR142, LYS167, ILE219, LYS220, and TYR274 contributed individual energies above the −1.0 kcal/mol threshold. For the TOP1+Quercitrin complex (Figure 5C), LYS133, THR142, ILE219, and TYR274 were found to contribute individual energies above the −1.0 kcal/mol threshold. For the TOP1+Ofloxacin complex, GLY132, LYS133, ASN140, THR142, LYS167, TYR209, and ILE219 contributed individual energies above −1.0 kcal/mol, respectively.

### 2.6. SPR Results

**Figure 6:**
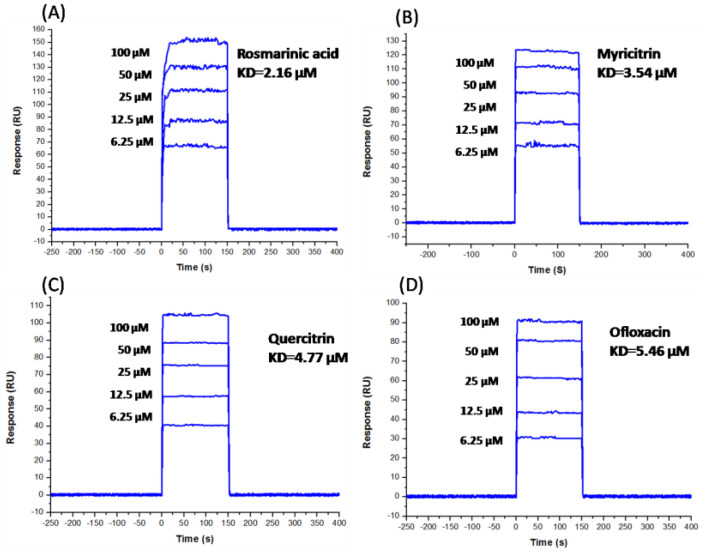
The direct binding affinity of selected compounds with vaccinia virus TOP1 was identified by SPR. (**A**) TOP1+Rosmarinic acid; (**B**) TOP1+Myricitrin; (**C**) TOP1+Quercitrin; (**D**) TOP1+Ofloxacin.

SPR experiments were applied to calculate the binding affinity between compounds and MPXV TOP1. Here, the binding affinity was measured with the equilibrium dissociation constant (KD). A wide range of half-diluted concentrations (from 6.25 to 100 μM) was employed to fit the KD values of four compounds (rosmarinic acid, myricitrin, quercitrin, and ofloxacin). A single-site model was used to fit compound half-diluted concentrations versus the response (RU). The KD of rosmarinic acid, myricitrin, quercitrin, and ofloxacin were also calculated as 2.16 μM, 3.54 μM, 4.77 μM, and 5.46 μM, respectively. The representative sensorgram of binding RU versus time is depicted in Figure 6.

### 2.7. Analysis of Feasible Targets toward Rosmarinic Acid through GO and KEGG Enrichment

**Figure 7:**
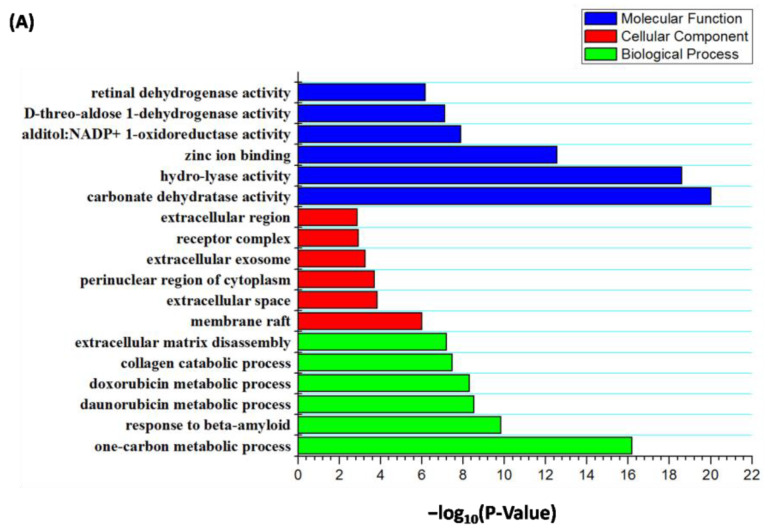

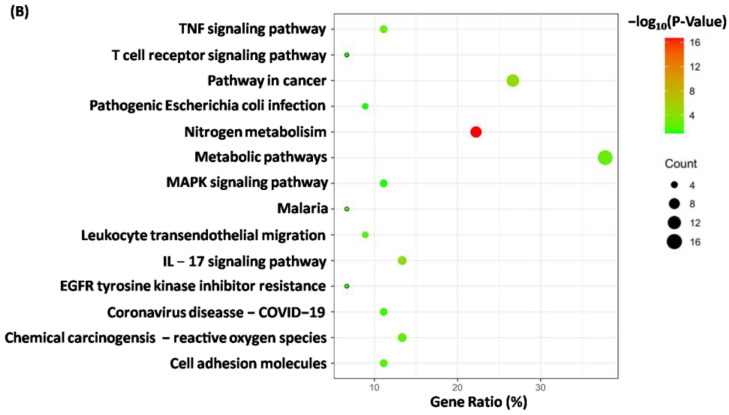
Analysis of the feasible targets toward rosmarinic acid through GO and KEGG enrichment. (**A**) The forecasted targets mostly take part in carbonate dehydratase activity in terms of the molecular function; as for the cellular component, the predicted targets mainly existed in cytosol; one-carbon metabolic is considered the primary progress during the biological process. (**B**) The circle diameter represents the number of rosmarinic acid-related genes. The deeper shield of orange shows the greater disparity. Forecasted genes towards rosmarinic acid (CA12, CA1, CA5B, CA2, CA4, CA7, CA6, CA9, CA14, and CA13) were allocated to the signaling pathway of nitrogen metabolism, with notable differences.

More than 100 target genes were yielded for rosmarinic acid using Swiss Target Prediction. Three enrichment branches were formed from the GO annotation output: molecular function (MF), cellular component (CC), and biological process (BP) (Figure 7A). Carbonate dehydratase and hydro-lyase activity were shown to be outstanding in rosmarinic acid-related MF. For CC, the rosmarinic acid-predicted target mostly took part in the membrane raft and extracellular space. The one-carbon metabolic process was thought to closely interplay with rosmarinic acid-predicted targets towards BP.

The KEGG pathway showed that nitrogen metabolism may be an important pathway towards rosmarinic acid targets (Figure 7B). These pathways are closely linked with innate and adaptive immune cells in the skin, indicating that the utilization of rosmarinic acid may allow for the suppression of inflammation during MPXV infection [21].

## 3. Discussion

Natural products, including TCM, have shown their potential to be repurposed as effective MPXV therapeutics. Here, we explored possible MPXV inhibitors by targeting TOP1. TOP1 is a topoisomerase with 314 amino acids, which recognizes and trans-esterifies at specific DNA sequences, such as 5′-(T/C) CCTT↓, where 30 phosphates of the incised strand bind with TYR274 towards the enzyme [22]. The amino acid at position 274 is the key amino acid towards the TOP1-binding pocket. Many enzymes are encapsidated in the orthopoxvirus. Topoisomerase I (TOP1) is a crucial enzyme required for the early phase of viral transcription. Therefore, TOP1 is considered a potential antiviral target [10]. However, the lack of a TOP1 tertiary structure in the recent outbreak of MPXV seriously narrowed the rational design of inhibitors. Therefore, a homology model was generated using the high-quality structural templates available from the smallpox virus, which shares 98.41% sequence identity with MPXV.

The novel TOP1 model identified three potential bioactive compounds comprising rosmarinic acid, myricitrin, and quercitrin with binding scores of −8.207 kcal/mol, −7.599 kcal/mol, and −7.322 kcal/mol, respectively. TYR274 was predicted to be a critical residue for those compounds anchored in the pocket of DNA TOP1 (Table 2, Figure 5). The selected compounds were demonstrated to have higher binding affinities than the clinical molecule (ofloxacin), with a docking score of only -6.046 kcal/mol. Furthermore, the potential inhibition of TOP1 by the compounds was corroborated through MD simulations, including MM/PBSA. Rosmarinic acid, myricitrin, and quercitrin demonstrated better affinity against the TOP1 of MPXV than ofloxacin, although ofloxacin was a typical inhibitor with defined targets in the vaccinia virus. To be more specific, a significant number of key residues (PHE131, GLY132, LYS133, THR142, LYS167, ASP168, LYS169, and TYR274) contribute favorably to the interaction with rosmarinic acid in comparison to ofloxacin (PHE131, GLY132, and ASN140). TYR274 was the unique residue that conferred individual energy that ranked top in the TOP1–compounds complexes. Thus, TYR274 means a lot for ligand binding with TOP1, which warrants studies for further validation and to identify its role. In this study, natural products from the TCM molecule library, which were identified to have desired binding affinities, deserve further experimental validation.

To explore whether the selected compounds could directly interact with TOP1, SPR experiments were administrated. The vaccinia TOP1 was chosen as the target protein in this study, because it presented high homology with monkeypox TOP1 (99%). A low equilibrium dissociation constant (KD) of rosmarinic acid, myricitrin, quercitrin, and ofloxacin was also calculated as 2.16 μM, 3.54 μM, 4.77 μM, and 5.46 μM, respectively. Figure 6 presents the binding affinity of TOP1 with rosmarinic acid, myricitrin, quercitrin, and ofloxacin, which increased with the level of concentration, and rosmarinic acid’s affinity to TOP1 ranked as having the optimum sensitivity compared to the other compounds (Figure 5). Taken together, the above outcome proves that TOP1 may directly target rosmarinic acid, myricitrin, quercitrin, and ofloxacin.

Recently, Merecz-Sadowska et al. proved that extracts from Leonotis nepetifolia, including rosmarinic acid, had a strong impact on topoisomerase I activity, leading to cytotoxic potential against human melanoma cells [23]. Soluble rosmarinic acid was originally isolated from rosmarinus officinalis and used as an active ingredient and index component from herbs and nutraceuticals in TCM. It is widely distributed in a variety of plants of Labiaceae, Sycamaceae, Pomeraceae, Tiliaceae, and Umbelliferaceae. A special caffeoyl moiety confers rosmarinic acid with antiviral properties. Previous reports revealed that rosmarinic acid inhibits several viruses, such as HPV, VSV Ebola-pseudotyped, and vaccinia viruses [24]. In addition, heparan sulfate proteoglycans-mediating cellular attachments are required for viruses that strongly interfere with the caffeic chelates [24]. The result from the targeted prediction of rosmarinic acid during the cellular component also supported the assumption that the predicted target mainly participates in the membrane raft and extracellular space (Figure 6A).

Many flavonoids from natural products used both as medicine and food have strong in vitro and antiviral efficacy [25]. Both myricitrin and quercetin are dietary flavonoids with few adverse effects, which present potential anti-MPXV properties in our study. Multiple computational and experimental studies revealed that flavonoids, especially flavonols and their derivatives, are effective viral inhibitors, as well as possessing significant anti-inflammatory activities [26,27].

The bioinformatics analysis was utilized to briefly discuss the feasibility of cellular biological processes and pathways towards rosmarinic acid. Carbonic anhydrases ranked highly as the most likely target towards rosmarinic acid (Figure 6A). A previous study showed that carbonic anhydrases mainly regulate the pH and osmotic balance. Suri et al. further demonstrated that carbonic anhydrase II plays a critical role in Th2-relayed and toll-like receptor 3-caused pathways in inflammatory skin circumstances [28]. The functional annotation of GO and the enrichment analysis forecasted that the signaling pathways may participate in the anti-inflammatory response with rosmarinic acid (Figure 6B). Interestingly, nitric oxide (NO) was highlighted as a crucial molecule in series signaling pathways, including the vascular, metabolic, immune, and antiviral pathways. The deleterious physiological situation caused by viral infection may be partially reversed through the restoration of normative NO levels via combined interventions, such as pharmaceutical, dietary, or complex behavioral interventions [29]. Therefore, possible MPXV inhibitors, such as rosmarinic acid, may be exploited by targeting TOP1 or restoring NO-associated inflammatory responses. Nevertheless, further studies are required to clarify the role of rosmarinic acid in mediating nitrogen metabolism during MPXV infection. Overall, we aim to investigate whether the anti-MPXV activity of rosmarinic acid not only depends on the inhibition of viral functional protein such as TOP1 but also the alleviation of host inflammation caused by NO.

Multiple reports showed that both myricitrin and quercitrin are potential natural antioxidant agents [30,31]. Recent research further revealed that rosmarinic acid, coupled with flavonoids, conjugates with antioxidant and anti-inflammatory properties [32]. The application of those natural antioxidant agents is expected to inhibit viral replication, as well as alleviate the oxidative stress caused by viral hyperinflammation. TCM contains promiscuous phytonutrients of rosmarinic acid, myricitrin, and quercitrin. However, there is still a gap in the knowledge of its bioavailability towards rosmarinic acid, myricitrin, and quercitrin and their clinical applications. More studies emphasizing compound metabolites are still required. Although several vaccines and drugs against MPXV are being highlighted for their efficacy, exploring the repurposing of natural products from TCM may provide alternatives against MPXV. In summary, grouping some of these phytonutrients into the right combination in the form of a food supplement not only boosts the immune system to prevent viral spread but also further suppresses hyperinflammation, providing both prophylactic and therapeutic support against MPXV [33].

## 4. Materials and Methods

### 4.1. Homology Modeling

The TOP1 sequences were obtained from the NCBI (https://www.ncbi.nlm.nih.gov/protein/YP_010377093.1?report=genbank&log$=prottop&blast_rank=5&RID=J7W751W701R/ (accessed on 19 December 2022)) with the accession code YP_010377093.1 or were obtained from UniProt with ID F1DIV3. The homology model of TOP1 was obtained using SWISS-MODEL (SWISS-MODEL, https://swissmodel.expasy.org/ (accessed on 19 December 2022)) [34]. The TOP1 amino acid sequences were submitted into SWISS-MODEL, which implemented the Basic Local Alignment Search Tool (BLAST) search to obtain reasonable templates that were identical to the target sequence. The TOP1 structures from the variola virus or vaccinia virus (PDB ID: 3IGC, 2H7F, and 1A41) ranked top, with identity scores of 96.15, 98.08, and 96.94, respectively. The three structures were then chosen as the structural template to model the MPXV TOP1 [35].

### 4.2. Structural Validation

SAVES v6.0 (UCLA-DOE LAB, (Los Angeles, CA, USA)) was applied to assess the quality of the producing models (https://saves.mbi.ucla.edu/ (accessed on 22 December 2022)). The reliability of the model was validated by analyzing its Ramachandran plot, as well as the measurement of the 3D model’s compatibility by the Verify 3D and PROCHECK services (https://saves.mbi.ucla.edu/results?job=1051156&p=procheck/ (accessed on 22 December 2022)) [11,35].

### 4.3. Prediction of Binding Sites

MOE 2015.10 (CCG, (Ottawa, CAN)) was applied to investigate possible binding sites of the TOP1 model protein, and the docking grid file centered on the cocrystal ligand at the active site was generated by the Receptor Grid Generation module of Schrödinger 2021 [36]. A total of 13 binding sites were predicted using MOE, and Hyd, which ranked top, was selected for further study. The selected Site Finder found 50, 1.96, 72, and 132 in terms of the sizes for PLB, Hyd, and Side, respectively. The site that ranked top was selected for further receptor grid generation. The site residues are listed as follows: ARG67; GLN69; VAL77; ARG80; ASN81; LYS83; ARG84; ARG86; ILE87; ARG90; PHE127; PHE128; ILE129; ARG130; PHE131; GLY132; LYS133; TYR136; ASN140; THR142; VAL143; GLY144; PHE164; GLY166; LYS167; ASP168; LYS169; HIS172; PHE174; TYR209; ILE212; ARG218; ILE219; LYS220; ARG223; THR224; VAL263; GLY264; HIS265; ARG272; ALA273; TYR274;

### 4.4. Preparation of Protein Targets and Ligand Libraries

The compounds were acquired from the Traditional Chinese Medicine Library and the Natural Product Library (https://www.selleck.cn/screening/natural-product-library.html/ (accessed on 23 December 2022)). Schrödinger Maestro was utilized to generate the necessary format of the ligands or receptor grid. A total of 4103 natural micromolecules in the format of 2D spatial data files (sdf) were uploaded to the LigPrep module process. The possible ionization states of the small molecules were generated in the specified PH range (PH = 7.0 ± 2.0) under the condition of an OPLS4 force field; then, all possible combinations of the chiral atoms were generated. Each ligand could generate up to 32 different conformations and generate one low-energy ring conformation. Eventually, the small molecules were transformed into 3D structures (maestro). In addition, the compound libraries were sifted according to Lipinski’s rule of five. Ofloxacin, which presented a strong inhibition of the vaccinia TOP1 poxvirus, was also included in this study [37].

The structure minimization step was done in the preparation of protein targets via the Protein Preparation Wizard module in Schrödinger 2021, including the addition of hydrogen atoms and residue sidechains, structure optimization, and energy minimization. Water was removed from the modeled TOP1, while other impurity molecules were removed from the model structure. The protonation state of the residues in the protein was generated under a specific PH condition (PH = 7.0). Finally, the system was optimized under the OPLS4 force field to prepare a more reasonable protein structure.

### 4.5. Receptor Grid Generation and Virtual Screening

Schrödinger was applied for the process of virtual screening. The Receptor Grid Generation module was used to generate the docking grid file centered on the binding site. The coordinate of the binding site was parameterized based on the MOE-binding site prediction. The virtual screening was based on three kinds of different precision screenings and MM/GBSA [36]: (1) high-throughput virtual screening (HTVS), (2) standard precision screening (SP), and (3) extra-precision screening (XP). Firstly, HTVS filtered 50% unfitted compounds; the remaining 50% matched compounds were further employed for SP. Secondly, the top 15% fitted compounds of SP with better docking scores were performed for XP screening. Eventually, XP highlighted the remaining 10% of the compounds with the desired docking score. Meanwhile, MM/GBSA was utilized to calculate the binding affinity between the compound and TOP1, respectively.

### 4.6. Characterisation of Binding Mechanism

The binding between TOP1 and the compounds was evaluated and analyzed via Glide docking (Maestro).

### 4.7. MD Simulations of Protein–Ligand Complexes and Proteins

The mechanism of the protein–ligand interactions was clarified through energy minimization in a precise solvent with AMBER18. The forcefields of ff14SB and GAFF were used for the protein and the small molecules, respectively. To be specific, the complex of TOP1–ligands was dissolved virtually in a rectilinear box of TIP3P3 water molecules buffering 6 Å from the macromolecular system, with a total amount of 17,990 immersed water molecules used for solvation. Energy minimization was implemented in the following two consecutive steps with the default parameters. Canonical ensemble (NVT) and isothermal-isobaric ensemble (NPT) were used for equilibrium system, and a MD simulation of 100 ns was performed at a normal temperature and pressure. The RMSD, RMSF, Rg, and SASA were employed to test the complicated stability after the project was completed [38]. The computation of the complexes was implemented using MM/GBSA, where the binding energy was calculated, and the individual energy contributions of the residues were found [38].

### 4.8. Surface Plasmon Resonance (SPR) Analysis

Four selected compounds, including rosmarinic acid, myricitrin, quercitrin, and ofloxacin, as well as the vaccinia DNA Topoisomerase I, were procured from Selleck (Shanghai, China) and Beyotime (Beijing, China), respectively. The SPR experiments were implemented on the system of the ProteOn XPR36TM SPR instrument (Bio-Rad, Hercules, CA, USA) [39]. Firstly, the vaccinia TOP1 was immobilized with standard amine coupling on the EDC/NHS and activated the GLH biosensor chip surface (Bio-Rad). A TOP1 solution of 1 mg/mL PBST (5 mM, 7.4 pH) was diluted to 30 μg/mL (pH 4.5). Then, TOP1 (5 μL/min for 400 s) was charged and covalently fixed. The final immobilization level for TOP1 was around 18,000 RU. The selected compounds were provided in PBS with 0.005% Tween-20 (pH 7.4) and injected at 20 mL/min for 150 s at concentrations of 6.25–100 μM (doubling dilution). The related phases with 5 concentrations towards 150 s were simultaneously injected at a flow rate of 30 mL/min, and then, the phases of dissociation were injected at 25 °C for 250 s. The chip surface was regenerated with 30 s pulses of running buffer after compound injection. The collection of data was reference-subtracted via ProtedOn ManagerTM 2.0. OriginPro 8 software was used for the data analysis.

### 4.9. Forecasting for Mechanism of Action towards Rosmarinic Acid

The structure of rosmarinic acid was submitted to Swiss Target Prediction (http://www.swisstargetprediction.ch/ (accessed on 3 December 2022)) to screen the potential target gene [40]. The functional annotation of the Database for Annotation, Visualization and Integrated Discovery (DAVID) v6.8 (Laboratory of Immunopathogenesis and Bioinformatics, (Maryland, USA)) was implemented to annotate the target gene, and the Official Gene Symbol was selected as the identifier in DAVID v6.8. Both Gene Ontology (GO) and the Kyoto Encyclopedia of Genes and Genomes (KEGG) were used to analyze each target gene [41]. The enrichment bubble plot of the KEGG pathway was formed by R program v3.5.0.

## 5. Conclusions

In this study, natural products from TCM molecule library were screened via Glide docking (Maestro). Firstly, the acceptable MPXV TOP1 structure was modeled according to the 3D structure of variola virus TOP1 via SWISS-MODEL due to the lack of an available structure in the Protein Data Bank. Subsequently, the virtual screening was based on three kinds of different precision screenings (HTVS, SP, and XP) and MM/GBSA. Three compounds (rosmarinic acid, myricitrin, and quercitrin) presenting with good docking scores and binding affinity were highlighted and purchased for further SPR measurements.

It can be assumed that three potential antiviral compounds, comprising rosmarinic acid, myricitrin, and quercitrin, were discovered through docking-based virtual screening. The following study clarified TYR274 as a crucial residue for compound binding affinities. MD simulations, including MM/PBSA, corroborated the potential inhibition of TOP1 by the compounds. SPR was applied to evaluate the binding affinity of the vaccinia virus TOP1 in vivo. Further experiments are needed to determine whether the compounds have inhibitory activity against MPXV TOP1. Additionally, most of the data provided in this paper were based on a computer-aided drug design. Therefore, the clinical application requires further evaluation of the possible inhibitors through experimental confirmation both in vitro and in vivo.

## Figures and Tables

**Table 1:** The chemical features and docking scores between TOP1 and selected compounds.

Molecule Name	Molecule Structure	Molecular Weight	Docking Score (kcal/mol)
Rosmarinic acid	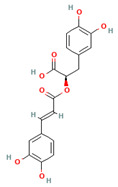	360.32	−8.207
Myricitrin	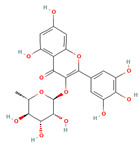	464.38	−7.599
Quercitrin	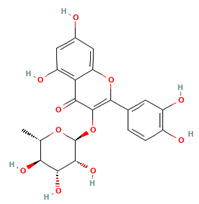	448.38	−7.322
Ofloxacin	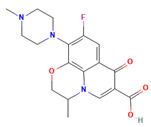	361.40	−6.046

**Table 2:** The MM_PBSA and hydrogen/hydrophobic bonds between the selected compounds and TOP1.

Compounds	MM_PBSA (kcal/mol)	Hydrogen Bond	Hydrophobic Bond
Rosmarinic acid	−16.18	TYR274, LYS167, GLY132, LYS133, ASP168	ARG223, LYS220 ARG130, PHE131 TYR136, THR142 ASN140, LYS169
Myricitrin	−13.87	TYR209, TYR274, LYS167, GLY132, LYS133	ARG218, ILE219 LYS220, ARG223 ARG130, PHE131 TYR136, THR142
Quercitrin	−9.40	TYR209, TYR274, LYS167, LYS133, GLY132	ILE219,LYS220, ARG223,ARG130, PHE131, TYR136, THR142
Ofloxacin	−9.64	PHE131, GLY132, ASN140	ARG67, ARG130, LYS133, TYR136, THR142, LYS167, ASP168, ILE219, LYS220

## Data Availability

Not applicable.

## Sample Availability

Samples of the compounds (Rosmarinic acid, myricitrin, quercitrin, and ofloxacin) are available from the authors.
